# Multi Clustering Recommendation System for Fashion Retail

**DOI:** 10.1007/s11042-021-11837-5

**Published:** 2022-01-13

**Authors:** Pierfrancesco Bellini, Luciano Alessandro Ipsaro Palesi, Paolo Nesi, Gianni Pantaleo

**Affiliations:** DISIT Lab., University of Florence, DINFO dept, Florence, Italy

**Keywords:** Recommendation systems, Clustering, Customer and items clustering composed

## Abstract

Fashion retail has a large and ever-increasing popularity and relevance, allowing customers to buy anytime finding the best offers and providing satisfactory experiences in the shops. Consequently, Customer Relationship Management solutions have been enhanced by means of several technologies to better understand the behaviour and requirements of customers, engaging and influencing them to improve their shopping experience, as well as increasing the retailers’ profitability. Current solutions on marketing provide a too general approach, pushing and suggesting on most cases, the popular or most purchased items, losing the focus on the customer centricity and personality. In this paper, a recommendation system for fashion retail shops is proposed, based on a multi clustering approach of items and users’ profiles in online and on physical stores. The proposed solution relies on mining techniques, allowing to predict the purchase behaviour of newly acquired customers, thus solving the cold start problems which is typical of the systems at the state of the art. The presented work has been developed in the context of Feedback project partially founded by Regione Toscana, and it has been conducted on real retail company Tessilform, Patrizia Pepe mark. The recommendation system has been validated in store, as well as online.

## Introduction

The competitiveness of retailers strongly depends on the conquered reputation, brand relevance and on the marketing activities they carry out. The latter aspect is exploited to increase the sales and thus a retailer, through marketing, should be capable to stimulate customers to buy more items or more valuable items. Today, consumers tend to buy more on ecommerce and the COVID-19 situation also stressed this condition. Online shopping offers the possibility to buy at any time of the day; customers buy where they find the best offer, online as well as offline, and they are also influenced by an increasing amount of information from blogs, communities, and social networks. To retain a customer is therefore an extremely difficult achievement, and in some measure, it can get easily out of control.

Currently, ICT (Information and Communication Technologies) offers Customer Relationship Management (CRM) solutions that are capable to construct and manage user data profiles, from customer information to product details, to sales transactions. CRM systems comprise a set of processes to support business strategies to build long term profitable relationships with customers [49]. Customer data and information technology (IT) tools form the foundation on which successful CRM strategies are built. Swift in [55] defined CRM as an enterprise approach to understand and influence customers’ behaviour through meaningful communications in order to improve customer acquisition, retention, loyalty, and profitability. However, CRM solutions on the market use approaches suggesting the most popular items, bundled offers, similar items or featured items and therefore they often neglect the relevance of customer personal preferences in their marketing strategies. In addition, there are IoT Devices offered by big vendors, promising an evolved engagement at various levels [28], interacting with less queues, promotions, more involvement, assistance, although they are hardly triggered within companies, especially on retail, which needs more flexible solutions. Therefore, market solutions are unable to build actual profiles by exploiting users’ historical, social, and behavioural activities. Through transactions, retailers can generate knowledge about their consumer's behaviour. In this context, one of the techniques receiving more attention from researchers to generate consumer knowledge, is machine learning, specifically clustering techniques. Clustering techniques are used to group customers by similarity. So that, retailers can tailor marketing actions more effectively with respect to the above-mentioned generic marketing actions. Understanding the reasons why consumers choose a specific item within the store is of extreme relevance for the retailer. In addition, knowing the consumer's needs through the factors that influence shopper’s decision-making process is important for the business of each single store. This is what recommendation systems are all about. Recommendation systems are applications that assist users in finding items (products, services and information) that should match their preferences / needs [56]. The generated recommendations are considered (i) *personalized*, in the sense that they have been generated for a user or a group of users, or, in the opposite to (ii) non-personalized recommendations (e.g., best-selling items, or selection of items), which are typically not addressed by research.

Recommendation systems at the state of the art do not solve typical retail problems. Most of the retail companies today have both online and physical store customers who are assisted in purchasing by shop assistants. With the GDPR (General Data Protection Regulation) rules [5], often the customer demographics are differently collected in different areas and shops, where different regulations are adopted. Deep learning methods, which are typically employed to improve accuracy, are hard to be adopted for the scarcity of data. For example, in fashion retail shops most of the transactions are anonymous and related to a single item; moreover, periodic acquisitions are performed every 8–12 months. This behaviour is mainly due to the high costs of the items and to seasonality aspects of most of the products. Regarding classification methods, the multichannel nature of retailers allows to provide data with different features and with many incomplete records, which are difficult to be exploited on most of the classic methods for recommendations. As for clustering methods, we registered the usage of RFM (Recency – Frequency – Monetary Value) [15], and LTM (Life-Time-Value) [37], where demographic values are taken as input without taking advantage of the typical intuition of deep learning about customer behaviour with respect to items. Another problem related to the fashion retail industry is related to the seasonality of most of the items. Their commercial life ranges from 6 months to 1 year.

In this paper, a recommendation solution in the context of fashion retail is proposed. The aim has been to solve the above-mentioned problems of cold start, computational complexity, low number of returns in the shops of fashion retails and long period for returning, the needs of more mediated interactions in the shops and more direct interactions online, and the effects of the seasonality of products. To this end, we realized a multi clustering approach by taking as input the RFM value of online and physical stores separately. To solve the problem of the products’ seasonality the items have been clustered taking into account multiple seasons. In addition, input data have been enriched with the customer behaviour towards the items. In order to solve the cold start problem of cluster-based recommendation systems, the association rules mining technique has been used to predict the purchase behaviour of newly acquired customers. The work presented in this paper has been developed in the context of Feedback research and development project co-founded by Regione Toscana, Italy, and by partners. Partners of the project have been VAR Group, University of Florence (DISIT lab, DINFO dept.), TESSIFORM (*Patrizia Pepe* trademark), SICETELECOM, 3F CONSULTING and CONAD (External partner). The studies illustrated in this paper have been conducted on retail company Tessilform: which is a fashion retailer owning online sales and many different stores in the world, mainly in Italy, the owner of Patrizia Pepe trademark.

The paper is structured as follows. In Section II, related work on recommendation systems is presented. The section also includes a comparative table. Section III describes the system architecture adopted in Feedback solution. In Section IV, the proposed recommender systems based on multi clustering is presented using a number of subsections. The solution allowed to prepare the recommendations in advance and consume them in real time when the conditions occur, or for stimulating the customer to return in the shop via email and when they access on Web. In Section V, the assessment and validation are reported. Conclusions are drawn in Section VI.

## Related work

Recommendation techniques can be classified into six categories according to the sources of knowledge they use: [47, 51], and more precisely: content, collaborative filters, demographic, knowledge, community and hybrid. In **Table ****1**, a comparative overview of the reviewed methods for recommendation systems in literature is reported.

**Table 1:** Comparative overview of the main related works on recommendation systems for retail

Paper	Features	Data	Domain	Technology	Results
A Two Phase Clustering Method for Intelligent Customer Segmentation [48]	Demographic variables, RFM and LTV	Iranian Bank, 38,254 ratings, 491 customers with 25 attributes	Bank industry	Clustering K-means Neural networks classification	The accuracy has not been quantitatively evaluated, but based on the results, the solution allows to make better decisions to create suggestions and to create marketing strategies
Product Recommendation based on Shared Customer’s Behavior [52]	RFM and LTV Shopping Basket	Chain of perfumeries (2012–2013); 3245 customers, 11,000 products	Retail	Clustering – Association rule mining	Increases 96% the average value of the sales when compared with based recommendation
Recommender Systems Using Support Vector Machines [44]	Ratings	EachMovie 1000 users with more than 100 movie ratings	Simulated	Support Vector Machines – Genetic Algorithm	Using McNemar's test, Support Vector Machines – Genetic Algorithm model shows better performance than the SVM and Traditional models
Collaborative Filtering Recommendation Algorithm Based on Item Clustering and Global Similarity [61]	Items, Users, Ratings	MovieLens 100 K ratings of 943 users on 1038 movies	Simulated	Clustering K-means	Can improve the accuracy of the prediction and enhance the recommendation quality
Collaborative filtering with the simple bayesian classifier [45]	Ratings	EachMovie (movie ratings): ratings of 2000 users on 1410 movies and JesterData: ratings of 3000 users per 100 jokes	Simulated	Bayesian Classifiers	F-measure 70.02%
Learning and Revising User Profiles: The Identification of Interesting Web Sites. [50]	words in web pages	40 tests	Different domains	Decision trees classifier	Benefit in selecting features that are relevant to the classification task
Fast algorithms for mining association rules in large databases. [2]	transactions	100 K transactions	Different domains	Association rule mining	Good computational performance
Image-Based Fashion Product Recommendation with Deep Learning [58]	Descriptive metadata or user reviews, visual information	Fashion1 dataset. dataset contains 11,851 images, and the texture attributes dataset contains 7342 images	Simulated retail	kNN—CNN – AlexNet [39] and batch-normalized Inception	not available
Personal recommendation using deep recurrent neural networks in NetEase [62]	Web page sequence, purchase history	e-commerce website www.kaola.com	retail	RNN	It extracts the common purchase patterns and shorten the purchase path for future users, reaching a compression ratio of 0.724127 and an accuracy of 0.331312
Sentiment-Aware Deep Recommender System With Neural Attention Networks [20]	Item textual reviews	Amazon Product Reviews ( # Users: 784,926, #Items: 141,786, #Reviews: 1,064,767), Yelp 2017 Datasets Challenge # Users: 169,257, #Items: 63,300, #Reviews: 1,659,678)	Simulated retail	Attention Network	This approach in terms of MSE outperforms other algorithms with a value of 0.850 compared to values in the range from 0.901 to 1.884 of the other algorithms
RecSys-DAN: Discriminative Adversarial Networks for Cross-Domain Recommender Systems. [60]	review	Amazon dataset in five categories	Simulated retail	AN	Through the calculation of MAE and RMSE it has been demonstrated that the proposed technique performs better than other algorithms (such as KNN or SVD)
Deep Reinforcement Learning for List-wise Recommendations [63]	Recommendations	ecommerce database of 100,000 recommendation sessions (1,156,675 item)	Simulated retail	Markov Decision Process + DRL	Through the calculation of Normalized Discounted Cumulative Gain metrics, demonstrating that it outperforms long-term recommendations
Personalized Recommendation for Online Retail Applications Based on Ontology Evolution [3]	User profile	No validation	Different domains	ontology-based	The ontology evolution technique in recommendation detects the behaviour of the user and give accurate and updated recommendations to each user
Personalized Recommendation System Based on Collaborative Filtering for IoT Scenarios [17]	ratings	Simulated in different dataset	Different domains	k-means, FCM, SLINK, SOM	The results show that clustering improves the recommendation accuracy
Online discrete choice models: Applications in personalized recommendations, Decision Support Systems [43]	User profile	Swissmetro data set	Transportation	Discrete choice models	Computationally efficient and empirically accurate

The **content-based approaches** recommend items by computing similarities among items and users through a set of features associated to them [7, 8]. For example, for a clothing item the considered features can be the group (shirt, sweater, T-shirt, etc.), colour, popularity, etc.; while for the users: demographic aspects, surveys answers, etc. In [59] a content-based recommender system that suggests the most suitable items after the creation and first login of a new user, taking into account the similarity with other users and the popularity of the items. The proposed solution showed how to solve cold start problems for new users.

The **collaborative filtering-based** approach is based on the historical data of the user's interactions with the items, either explicit (e.g., user’s ratings) or implicit feedback (e.g., purchase, visit, tests). The mathematical techniques used are the neighbourhood method and the latent factor model [38]. The neighbourhood method identifies relationships among elements or, alternatively, among users. The latent factor model sets a number of evaluation methods to characterize both items and users and it is mainly based on the matrix factorization (for example the ratings-matrix). These kinds of approaches do not need a representation of the items, as they are based only on ratings, so they are the best recommendation systems in terms of scalability, since they act on rules or patterns instead of the entire dataset. The accuracy of recommendations increases as the user interactions increase. They have cold start problems for both new items and new users.

The **Demographic-based** approaches generate recommendations on the basis of the user's demographic profile (age, gender, education, etc.). They do not require a user ratings history, and they have cold start problems for new items. In [25], demographic information has been used to predict the number of products sold in a store and as a recommendation system. The experimental predictive accuracy was 1.5–5 times greater for the items of interest, as measured by r-squared error statistics.

The **knowledge-based** approaches are based on the knowledge of item features which meets the users’ needs. They do not have cold start problems; however, they require a broad knowledge of the domain and, in case of many items, they are very difficult to implement. In [32] a knowledge-based recommendation system has been implemented starting from the logs (purchase times and choices) of an ecommerce. The obtained results confirm an optimization of purchase times of purchase for customers.

The **community-based** systems make recommendation through the preferences of users' friends in contexts of social networks or communities. The basic concept is that a user tends to rely on recommendations from their friends instead of those of similar but unknown users. This approach is very useful for cold-start recommendations. In [23], a method is proposed to solve cold start problems in a recommendation system to suggest movies, which exploits the implicit relationships among the items derived from the direct interactions of the users with them.

The **hybrid-based** recommender systems combine two or more of the above listed recommendation approaches in different ways. Usually, considering two different approaches, the advantages of the former are used to mitigate the weakness of the latter. In [21], it was shown how a hybrid approach (demographic and collaborative filtering) improves the accuracy of item evaluation predictions compared to individual approaches.

The sources of knowledge are usually represented by three types of descriptors for: items, users and transactions (relations between user and item). Modern recommendation systems also use textual reviews [20], images [58], web page sequences [62], user emotion (Facial Expression Recognition [41] or even text reviews [19]), images and web page sequences, and processed through data mining or deep learning methods, to generate recommendations.

The data mining methods for recommender systems can be summarized in three types of algorithms, as follows.

**Classification**. For example, the kNN classifier finds the closest *k* points (closest neighbours) from the training records. In [10], kNN has been implemented to suggest short-term news to users. with very good results in terms of precision. Decision Trees classifier works well when objects have a limited number of features. In [50] and [18], it has been shown that this technique can have low performance, since small changes involve recalculating all distances between items or customers. In [16], the classification approach has been used for the identification of target customers minimizing the recommendation errors, by selecting users to whom the recommendations should be addressed, according to which categories of purchases they have made in a selected period of time. In [10], a Naive Bayes classifier has been used to predict the user's long-term preferences in the news domain, with excellent results in accuracy. Support Vector Machines (SVM) classifier is used to find a linear hyperplane (decision boundary) that separates input data in such a way that the distance among data groups is maximized [44].

**Cluster Analysis** has been used for segmenting a heterogeneous population into a number of subgroups [6, 9]. Through the Clustering Analysis, it is possible to explore the data set and to organize the data for creating recommendations. For example, variables used in the clusters may be: demographic [25], RFM [15], LTV [37], demographic + RFM [35], demographic + LTV [33], LTV + RFM [14]. The commonly used clustering algorithms are: K-means (each cluster is represented by the geometric centre of the data points belonging the cluster, supposing the feature on some numerical space); K-Medoids (each cluster is represented by the most representative element of the cluster); Clara (it is an extension to Partitioning Around Medoids, PAM, adapted to large data sets); Self-Organizing Map (SOM, it is based on artificial neurons clustering technique) [48, 61]. About the Internet of Things (Iot) context in [17] k-means, fuzzy c-means (FCM), Single-Linkage (SLINK), and Self-Organizing-Maps (SOM) techniques are used to manage sparsity, scalability, and diversity of data in different domains. The results show that clustering improves the recommendation accuracy.

**Association Rules** aim at finding rules in the dataset that satisfy some minimum support and minimum confidence constraints. An association rule is an expression *X ⇒ Y*, where *X* and *Y* are item sets (e.g., Milk, Cookies ⇒ Sugar). Given a set of transactions *T*, and denoting MinSup and MinConf the minimum support and the minimum confidence constraint values, the goal of association rule mining is to find all rules having support greater than or equal to MinSup, and confidence greater than or equal to MinConf. The most common algorithms used for implementing association rule mining are apriori [1], FP-Growth (Frequent Pattern Growth) [29], SSFIM (Single Scan for Frequent Itemsets Mining) [22], and SETM (Set-oriented Mining) [31].

In [52], a **hybrid recommendation** system combining content-based, collaborative filtering and data mining techniques has been proposed. The recommendation algorithm makes similar groups of customers using LTV value, for this the segmentation of customer based on costumer behaviour through RFM attributes has been performed.

**Discrete choice models** are used to personalize recommendations as in [43] where a framework for estimating and updating user preferences in a recommendation system is presented. The authors demonstrated that the framework is computationally efficient and empirically accurate, however, parameter estimation can be inaccurate in the presence of non-heterogeneous data.

With the growing volume of data acquisition, the possibility of using **deep learning** in recommendation systems have been also considered, in order to overcome the obstacles of conventional models listed above, achieving a higher accuracy of recommendation. Through deep learning it is possible to detect non-linear and non-trivial relationships among users and items from contextual, textual and visual inputs [46]. The main limitations of deep learning-based recommendation systems are represented by the fact that there are often privacy issues in the collection of information for content-based systems, while for collaborative filtering the acquisition of data from different sources often results in incomplete information that greatly affects the accuracy of recommendations. The main deep learning algorithms for recommender system are described as follows. **Multilayer Perceptron** (MLP) is a class of feedforward artificial neural network with multiple hidden layers between the input and the output layer. In [30], a standard MLP approach to learn interaction among user and item latent features has been used by providing the model with flexibility and non-linearity. **Autoencoders** (AE) represent an unsupervised model that generate an output by compressing the input in a space of latent variables. There are many variants of autoencoders; the most common are denoising autoencoder, marginalized denoising autoencoder, sparse autoencoder, contractive autoencoder and variational autoencoder [54]. **Convolutional Neural Networks** (CNN) are feedforward neural networks that use convolution in place of general matrix multiplication in at least one of their layers. They can capture the global and local features and improve the efficiency and accuracy [58]. They have been used in several implementations, such as AlexNet [39] and batch-normalized Inception [34]. **Recurrent Neural Networks** (RNN) are typically employed to trace dynamic temporal behaviour, actually in this kind of neural network the connections among the nodes form a direct graph along a temporal sequence [62]. Other fields of research have achieved an improvement by exploiting **Long-Short Term Memory networks** (LSTMs) that minimize RNN problems regarding the gradient vanishing/exploding. LSTM have been applied in [57] to a movie recommendation system, in order to take into account users’ dynamic and time varying behaviour, and not only their static preferences. **Adversary Network** (AN) is a generative model where two neural networks are trained simultaneously within a minimax game framework [60]. **Deep reinforcement learning (**DRL) combines deep learning and reinforcement learning that enables to learn the best possible actions to attain the expected goals [63].

An **ontology-based** recommendation system has been proposed in [3]. The proposed architecture sends semantic recommendations for each user profile by applying content-based filtering and collaborative filtering techniques.

Compared to the previously discussed data mining techniques, all deep learning algorithms have cold start problems and require a considerable amount of data to improve performance. Open problems in the literature for deep learning-based recommendation systems concern the frameworks scalability and the explicability of generated recommendations. On the other hand, deep learning solutions are not applicable in this case, in which the number of acquisitions per user is low, which is one of the most critical problems of fashion retail.

## System Architecture

In the context of fashion retail, the shops are typically small in size (they are also known as boutiques), and the customers in the shops are directly followed step by step by the attendees who provide suggestions and are ready to support them on every aspect. A similar scenario may occur on the online shopping, in which an online assistant is ready to follow the customer, while the customer can more easily ignore the pressions of the attendee. In both cases, the user profiles are improved with new data in the few occasions in which the customers interact, and thus the customers might be continuously engaged with suggestions. This is obviously not possible since it cannot be acceptable by all the customers. So that, a moderated engagement tool has to be provided that may consume the possible recommendations by proposing them directly to the customers (via some devices into the shops or online) or via the assistant. Thus, the suggestions can be provided only a limited number of times per experience, and in specific conditions to avoid annoying / irritating the customer.

**Fig. 1:**
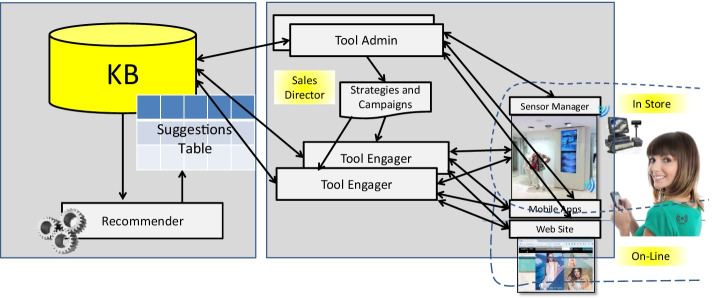
General Architecture

The architecture of the proposed system is reported in **Fig. ****1**. In compliance with GDPR rules, the Tool Admin stores the details of customers’ profile, items and transactions on stores and on the ecommerce website in a centralized database based on MS Azure. The Recommender reads the information from the KB (Knowledge Base) and generates the recommendations which in turn are stored in the Suggestion Table. The Tool Engager is the only responsible for sending recommendations to the customers, directly or via the shop assistant within the store. After that a recommendation has been sent, the Tool Engager has the duty to records the customer's interaction / reaction with respect to the recommendations (e.g.: detect and track if the customer reads the recommendation, accept the suggestion, test the product and eventually buy it). The Tool Engager are instantiated one for each shop or group of shops. The Recommender creates a list of suggestions taking into account users’ profiles and items’ descriptions, as described in the following. The recommendations have to be carefully provided, since suggested items should not have been purchased by the customer recently, neither already proposed by the human Assistant. All the suggestions need to be generated on the basis of purchases made by the customer in the last few experiences and months, when possible. These last rules for filtering are applied directly at the final stage by the Tool Engager, and this means that the provided suggestions have to be abundant with respect to those strictly needed to be consumed in short time, to be sure that the Tool Engager would have always new suggestions to be spent when it can be in conditions to deliver one. For example, when a newsletter is sent, the customer arrives on web shops, goes in the test room, etc. The Sensor Manager is capable to manage and collect data and events from sensors in the shops, such as those in the fitting rooms, close to totems in stores, RFID technology on items for proximity and customers’ interactions with products. All these data and events are identified and stored, and also trajectories performed by customers into the shop, which are tracked by using a Wi-Fi network of sensors. A rule system is capable to identify specific conditions to be sent to the Tool Engager, which enters in action providing or not a recommendation on the basis of discourse in place with the shop assistance and former suggestions. This paper is focussed on the Recommender.

## The Recommender System

**Fig. 2:**
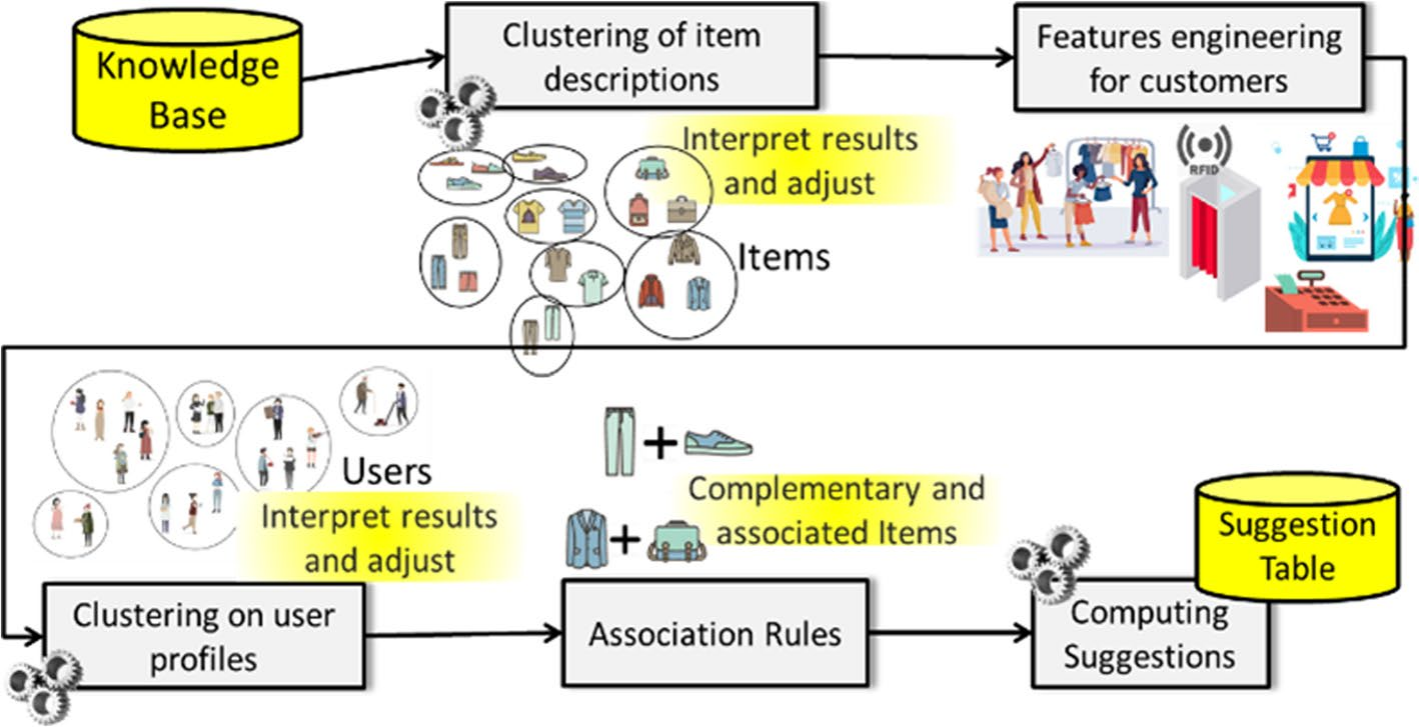
General Data Computing workflow

One of the main goals consisted in increasing the customer recency, and thus to increase the number of times users contacts and sales may occur. For this purpose, the computational workflow reported in Fig. 2 has been adopted. The data are continuously collected by the Tool Admin and Tool Engager (sales in shops: online and onsite) into Knowledge Base (see Fig. 1); then a periodic clustering on items is performed. The results are taken into account in the computation of an integrated clustering driven by the user profiles and additional features to finally provide a set of suggestions of different kinds. The main steps of the workflow are described in the following subsections.

The production of recommendations and their submission are asynchronous: (i) mediated by the assistant that may decide or not to accept and pass them to the customer, (ii) filtered by the Tool Engager according to the last actions performed by the customer, (iii) decided to be spent by sending them online via email when the time passed since the last contact with the users is greater than a reference value, or when the new products which fit with the user preference would be available, etc. The produced pool of recommendations (for each potential returning user, and user kind) is generated on the Suggestion Table, which is refilled on demand of the Tool Engager or periodically with a high rate. The Suggestion Table includes a programmed mix of suggestions computed by *customer similarity, items similarity, and serendipity (randomly produced).*

### Clustering of Item Descriptions

As above described, the first analysis has been performed to clusterize the item domain on the basis of their descriptions. This allows to reduce the space of all combinations and to weight the relevance of item categories. In the case of fashion retail, typically the number of products is not huge, differently to what one may have on supermarkets, in which a huge number of products is active on marketing at the same time. In our case of fashion retail, the database contained about 50,000 items which have been classified according to the fields reported in **Table ****2**, and which may belong to more than one season.

**Table 2:** Product Item descriptions Fields

Field ID	*Item Description*	*Example*
TYPE	Type	“1A0145”, “1A0333”,…
CONFIGURATION	Configuration	“DRESS”,“JACKET”,…
PATTERN	Color	“White”, “Red”, “Navy blue”, …
MODEL	Alphanumeric code model	“1A0145”, “1A0333”, …
PACKAGING_TYPE	Type packaging	"Packaging Basic PE", "Packaging Basic-Contin.", "Women's Packaging A/I",
PRODUCTION_CATEGORY	Production category	"Accessories", "Clothing", "Jeans", ……
MERCHANDISE_MCR_TYPE	Merchandise type	“Basic, Preview”, “Women”, “Main Women”, ……
MERCHANDISE_TYPOLOGY	Merchandise typology	“Preview Women SS”, “Main Women AI”, “Women PE”, ……
MERCHANDISE_MCR_FAMILY	Merchandise family	“Coat”, “Bag”, “Dress”, ……
MERCHANDISE_GROUP	Merchandise group	“Jewellery”, “Dress”, “Shirt”, ……
GENDER	Gender	“Accessories Women”, “Child”, “Women”, ……
BRAND	Brand	“VA”, “GM”, “PW”, ……
STYLE_GROUP	Style	“P”, “C”, ……
BIRTH_SEASON	Season	“20,201”, “20,062”, “20,071”, ……
PERIODICITY	Periodicity	“C”, “S”, ……
IS_CLOTHING_ITEM	Marking if the item belongs to a clothing category	1,0 (Yes/No)
NRM_CAT_LVL_1	Code normalized business classification level 1	“Accessories”, “Clothing”, “Jeans”, ……
NRM_CAT_LVL_2	Code normalized business classification level 2	“Bag”, “Clothing”, “Coat”, ……
NRM_CAT_LVL_3	Code normalized business classification level 3	“Shopping”, “Dress”, “Jacket”, ……
NET_SOLD_PRICE	Price	1580.00
IN_STOCK	Whether an item is available or not	1,0 (Yes/No)
132 X Hashtag tasche, abalze,…	Hashtag website	1,0 (Yes/No)

**Fig. 3:**
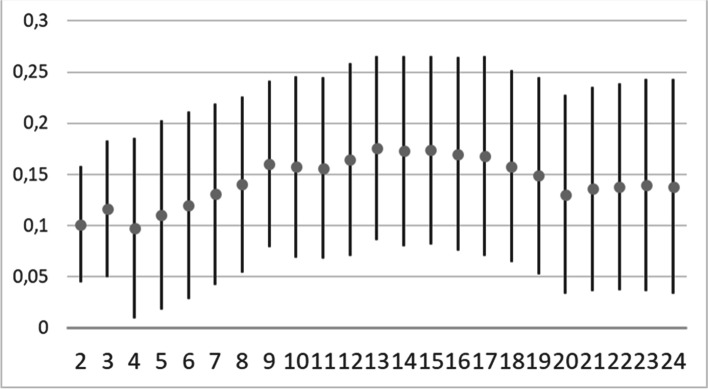
Trend of the Silhouette value as a function of the number cluster K for item dataset

Most of the fields are textual descriptions, thus they are strings coding the description; then, only a few of them provide numeric or Boolean. Therefore, the clustering cannot be based on Euclidean space. For this reason, the clustering has been carried out by using *K*-medoids [36], which is a classical clustering technique that partitions a dataset of *n* objects into *k* a priori known clusters. A number of techniques to identify the best compromise on the value of *K* can be used [42]. To calculate the distance among items we used the Gower distance [26], which is computed as the average of partial dissimilarities across individuals. Each partial dissimilarity (and thus the Gower distance) ranges in [0,1].

$$d\left(i,j\right)= \frac{1}{p}{\sum }_{i=1}^{p}{d}_{ij}^{(f)}$$

where: ${d}_{ij}^{(f)}$ is the partial dissimilarity computation which depends on the type of variable being evaluated. For a qualitative assessment, the partial dissimilarity is 1 only if observations *x*_*i*_ and *x*_*j*_ have different values, and 0 otherwise. Through the silhouette method we determine the optimal number of clusters. The silhouette method calculates the average silhouette of observations for different values of *K* [42]. The optimal number of clusters *K* is the one that maximizes the silhouette over a range of possible values for *K*. In **Fig. ****3**, the trend of silhouette index with its standard deviation as a function of *K*, is reported. From the trend, the value of *K* = *13* corresponds to the maximum of the averaged silhouette. It has been estimated as a compromise since the standard deviation is quite large, and a smaller number of clusters would provide too large sets for making selections.

**Fig. 4:**
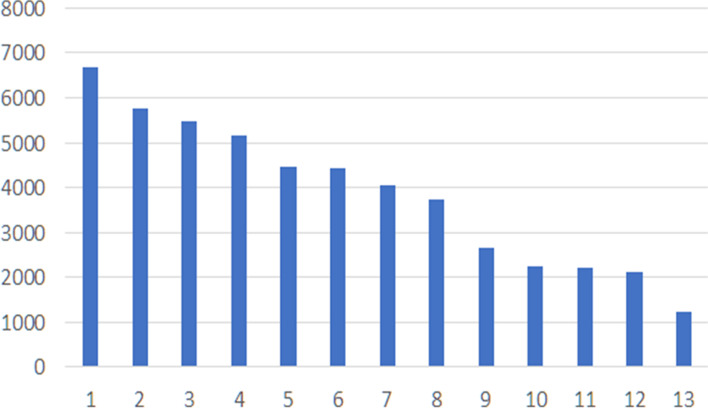
Distribution of size of the clusters in terms of items for K = 13

**Figure ****4** shows the distribution of clusters’ size for *K* = *13*.

In **Table ****3****,** the descriptions of the identified clusters, and the corresponding sales are reported. The main descriptions have been identified by a cluster analysis. The main drivers for clustering have been CONFIGURATION, MERCHANDISE_TYPOLOGY, BRAND and NRM_CAT_LVL_1.

**Table 3:** Main description of products’ clusters

Cluster	*Derived descriptions of the item clusters*	*# items*	*# sales*
2	DRESS, PE, clothing	6074	1171
1	BAG, AI, Accessory	6801	969
7	SHIRT, SS, Clothing	4346	838
3	TROUSERS, PE, Clothing	5786	794
4	KNIT, FW, knitwear	5222	678
5	T-SHIRT, PE, clothing	5100	674
6	ACCESSORIES (HAT—FOULARD—SCARF—NECKLACE—GLOVES—BRACELET), AI, Accessories	4479	596
10	SKIRT, PE, Clothing	2374	530
8	COAT, AI, Clothing	3133	388
9	SHOES, AI, Shoes	2835	341
11	JACKET, AI, Clothing	2365	292
12	BELT, AI, Accessory	2025	237
13	CHILDREN'S CLOTHING, Outlet PE. Cloting	1220	126

### Features engineering for customers

The data collected by the administrations and the retail shops refer to the user behaviour, which is associated with the user profile. The user profile is enriched with information regarding customer behaviour such as: (i) fields about the customer's maximum interest for an item within the cluster, such as: Interest (Yes/No), Observed (Totem, Online, etc.), Tried, purchased item; (ii) fields describing the items purchased within the cluster. Point (i) is a vector of 13 elements (one for each item cluster) where 0 identifies no interaction for the client with items in the cluster; 1 if at least one item in the cluster was observed by the client; 2 if at least one cluster item has been tried or placed in the shopping cart; 3 if at least one cluster item has been purchased. Point (ii) is a vector of 13 integers (one for each item cluster) which represents the number of items purchased by the client within the cluster, 0 if no item has been purchased.

In addition, a number of features (which in some sense are KPI, Key Performance Indicators) have been also computed, and assessed by taking into account the experience of business developers. Among them: recency, frequency, and average spending. *Recency* is defined as the number of days passed since the last visit or access in a store or online; *Frequency* represents the frequency of purchase in terms of the number of days; *Average spending* is the average value of single ticket for the customer (estimated on the basis of the admin track record). In addition, in order to distinguish from online and in-store behaviour, online and in-store frequency and recency are separately computed.

### Clustering on user profiling

In this case, the number of user profiles has been 608,447, of which 27,346 have been acquired in the 2016–2019 temporal range. The user profile includes the features listed in **Table ****4**. The features are the following: RFM_TRN_DaysFrequency is the frequency transaction, more precisely, how often the customer makes a transaction; RFM_TRN_DaysRecency is recency transaction, more precisely, how many days have passed since the customer's last transaction; RFM_TRN_AvgAmount is the average spending in a single transaction; RFM_PRS_ONLINE_DaysFrequency is the frequency presence online; RFM_PRS_ONLINE_DaysRecency is the recency presence online; RFM_PRS_ONPREM_ DaysFrequency is the frequency presence in a store; RFM_PRS_ONPREM_ DaysRecency is the recency presence in a store, FidelityUsageRange is fidelity card use, ranging from0 (lowest usage frequency 3 ( (highest usage frequency); CUS_FIDELITY_CARD_LEVEL_CD is the fidelity card level based on fidelity points accumulated according to the spending (0 is the lowest level, 3 is the highest); Cluster_k_Interest size [26] is the maximum interest in an item within the cluster; Cluster_k_Purchased size [26] is the number of items purchased within the cluster. Other features such as Gender, Age, Family Status, Fidelity card level, family status, country, city was not considered because, due to different sources of profile collection, they had incomplete or missing data, or they are constant in almost all records.

**Table 4:** 35 User customer features (all numbers)

Name profile feature	*Description*
RFM_TRN_DaysFrequency	Frequency transaction
RFM_TRN_DaysRecency	Recency transaction
RFM_TRN_AvgAmount	Average spending transaction
RFM_PRS_ONLINE_DaysFrequency	Frequency presence online
RFM_PRS_ONLINE_DaysRecency	Recency presence online
RFM_PRS_ONPREM_DaysFrequency	Frequency presence store
RFM_PRS_ONPREM_DaysRecency	Recency presence store
FidelityUsageRange	Fidelity card use
CUS_FIDELITY_CARD_LEVEL_CD	Fidelity card level
Cluster_k_Interest size [26]	Max interest for each cluster
Cluster_k_Purchased size [26]	Number of items purchased

**Fig. 5:**
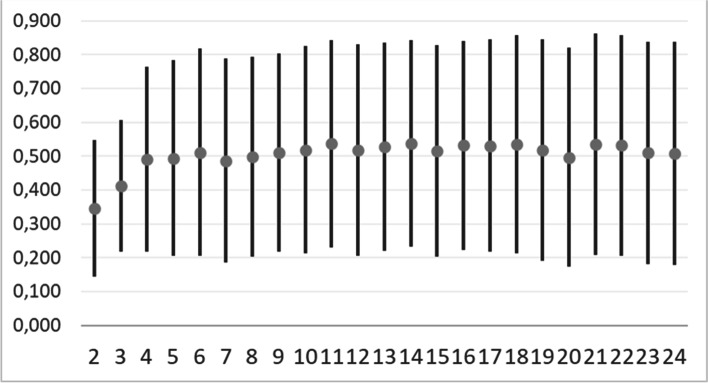
Average silhouette and its standard deviation vs number of clusters K

On the basis of the user profile features, which include two arrays of use’s preferences for items clusters identified in the first phase, a clustering has been carried out through the K-means method since the domain was Euclidean in this case. The Silhouette method has been used to determine the optimal number of clusters, in this case *k* = *14* (see **Fig. ****5**), taking the maximum of the average silhouette. The standard deviation is very large, while taking a smaller number of clusters would result in having too large clusters on which to make selections.

In **Table ****5**, the derived descriptions of customers/user clusters and they corresponding size are reported. Please note that the main features characterizing the clusters have been: average amount of spending, frequency and recency.

**Table 5:** Description of users’ clusters

Cluster	*Derived Description from Customer cluster analysis*	*# total customer*
1	Customers with average spending amount not defined; the frequency is not defined neither in store neither online; day of the last purchase not defined	9195
2	Customers with low average spending amount, mainly online with undefined frequency and last purchase older than two years	3158
3	Customers with undefined average spending amount, mainly in store, with undefined frequency and last purchase older than two years mainly online	2433
4	Customers with low average spending amount, last purchase older than one year	2302
5	Customers with low average spending amount in store, with frequency of about 4 months in store; last purchase has been made within one year. often using the fidelity card	2302
6	Customers with low average spending amount, more frequent in store with annual frequency; last purchase older than one year	1657
7	Customer with low average spending amount, more frequent online, but also buying in store with frequency of about 2 months online and about 6 months in store; last purchase older than one year, use fidelity card	1493
8	Customer with average spending amount not defined, mainly online; last purchase midterm days	1186
9	Customer with very high average spending amount in store	887
10	Customer with medium average spending amount more frequent in store but also buys in store with frequency about 230 days; last purchase about 262 days, use fidelity card	819
11	Customer with average spending medium amount in store; last purchase one year ago; frequency is not defined	797
12	Customer with average spending amount not defined, mainly online, with frequency of about 270 days; last purchase one year	717
13	Customer with medium average spending amount, mainly in store, with not defined frequency and last purchase older than one year	391
14	Online customers with annual frequency	90

**Fig. 6:**
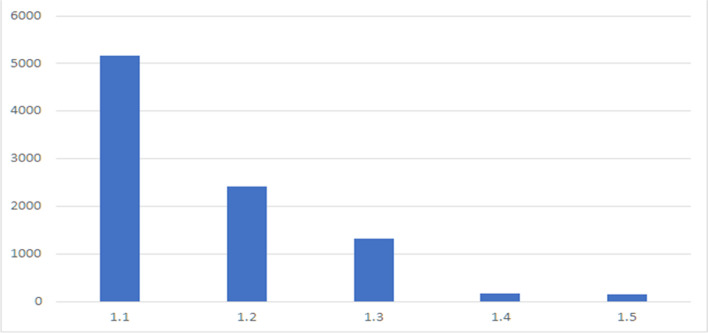
Distribution of customers along the resulting 5 clusters

According to the obtained results, cluster #1 was actually very large. For this reason, a second level clustering has been performed to split user cluster #1 in subclusters based on the same features. The Silhouette method has been used to determine the optimal number of clusters. The clustering result was initially highly unbalanced regarding the customers distribution, therefore a further analysis on distributions of customers at varying cluster size led to take *K* = *5*, with the aim of having maximum classifications and expression, as shown in **Table ****6**. The distribution of clusters has been reported in **Fig. ****6**.

**Table 6:** Description of second level cluster of cluster #1

Cluster	*Derived Description from Customer cluster analysis*	*# total customer*
1.1	Customers with average spending amount undefined; the frequency is undefined neither in store nor online; day of the last purchase undefined	5133
1.2	Customers with low average spending amount. They mainly buy in the product cluster #12	2411
1.3	Customers with very low average spending amount, mainly in the product clusters: #2, #10 and #12	1330
1.4	Customers with recency of about 23 days, frequency of about 18 days	173
1.5	Customers with average spending amount of about 150 Euro; mainly buying in the product cluster #1	148

**Fig. 7:**
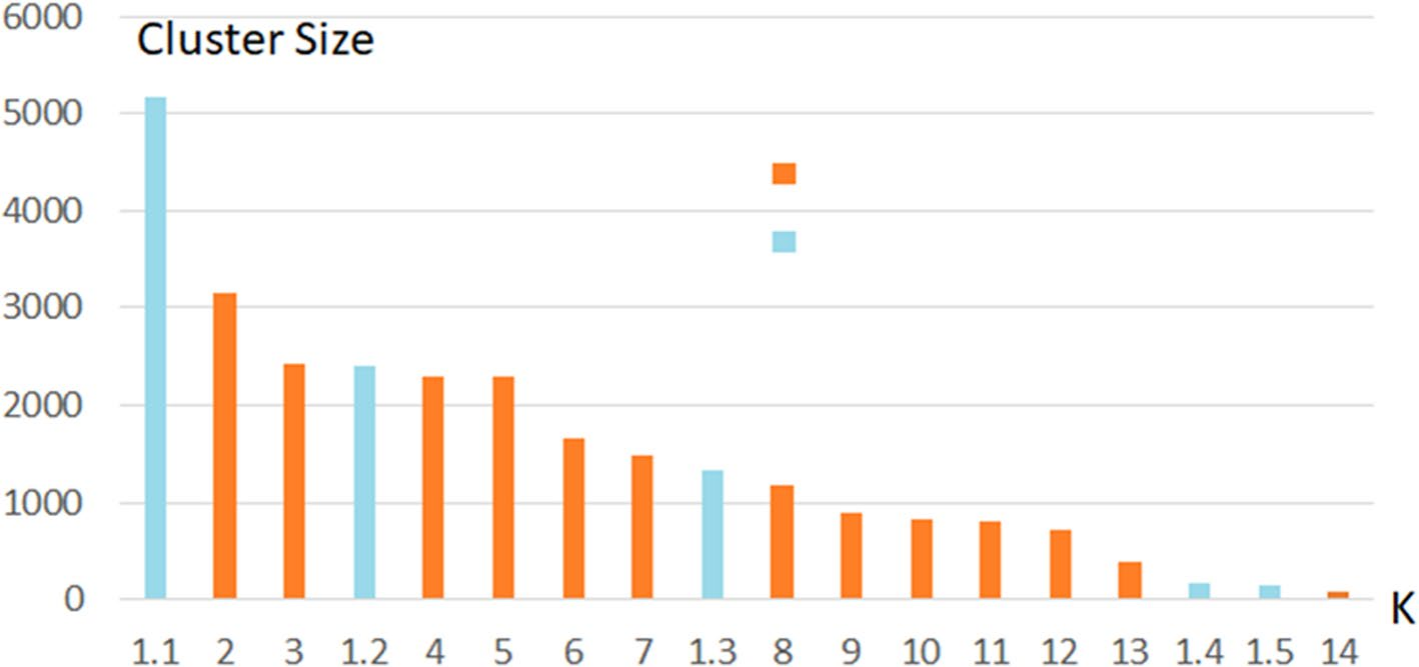
Distribution of 18 clusters for the number of customers, including first level cluster from 2–14 and second level from 1.1 to 1.5

The final distribution of clusters has been reported in **Fig. ****7**. In which the first level clusters are numbered from 2 to 14, and those of the second level clustering decomposing cluster 1 (of 9195 units) has been decomposed in clusters from 1.1 to 1.5.

### Computing Suggestions

As described above, the identified solution produces a number of recommendations for each user. Each possible suggestion is labelled with the kind, the date of emission, and a deadline. The Engager Tool also marks those that have been spent with the date and time of emission, the channel adopted (shopID, mobileApp, website, shopID. totemID, etc.), the ID of the assistant, etc. This information is useful for the assessment of the acceptance level at follow up, and thus for the validation, as described in the next Section. Therefore, the database with the suggestions is never discharged since the recommender must take into account the already spent suggestions.

The recommendations are generated according to different kinds (as described in the following list) and they are consumed in different contexts by the Engager Tool. Thus, the rate and the percentage of their exploitation/consumption depend on the decisions of the Engager and on the number of occasions in which the recommendations can be provided. Moreover, since the number of suggestions is abundant, they are also substituted with new ones if not consumed in a reasonable time. The different kinds of recommendations are by:

**customer similarity:** for each customer cluster the most representative items are computed. They are identified among the most purchased items within the users’ ones belonging to the same item cluster (they can be selected by using other criteria, for example: because they are the most frequently asked, or the company would like to push them, or they are closer to the cluster centroid, or to maximize the revenue or minimize the stock, etc.). In addition, the suggested item should have not been already purchased or proposed/suggested to same customer in the same season.

**item similarity:** considering the last items purchased by the customer according to the information contained into its profile, and randomly selecting items in the same item clusters, avoiding proposing items which have been already bought or proposed. Also in this case, the items can be filtered/selected by using additional criteria, for example: because they are the most frequently asked, or the company would like to push them, or they are closer to cluster centroid, or to maximize the revenue or minimize the stock, etc.

**item complementary:** considering items that may complement the last items that have been bought by the customer according to a table of complementary items; for example: a belt in combo with a bag. Please note that some of the item clusters are complementary each other, see the above descriptions – e.g., #1 and # 2 of Table 3. To this end, through association rules using a priori algorithm [1] for each transaction in the dataset a set of metrics have been calculated; some examples are reported in Table 7, for the first 5 clusters.

**Table 7:** Example of complementary clusters assessment by using metrics: support, confidence, lift and count (part)

Cluster	Complementary clusters				
	*cluster*	*Support*	*Confidence*	*Lift*	*Count*
1	2	0.26486066	0.6069351	1.106003	12,935
	7	0.24864345	0.5697729	1.253423	12,143
	3	0.24465057	0.5606231	1.213722	11,948
	8	0.24336057	0.5576670	1.277549	11,885
	4	0.22298667	0.5109797	1.282096	10,890
2	3	0.34351004	0.6259701	1.355196	16,776
	7	0.32391425	0.5902612	1.298495	15,819
	8	0.31392182	0.5720522	1.310504	15,331
	4	0.29840080	0.5437687	1.364367	214,573
3	2	0.34351004	0.7436830	1.355196	16,776
	7	0.30397035	0.6580814	1.447690	14,845
	8	0.29868747	0.6466442	1.481385	14,587
	4	0.27753548	0.6008511	1.507592	13,554
	1	0.24465057	0.5296569	1.213722	11,948
4	2	0.29840080	0.7487156	1.364367	214,573
	3	0.27753548	0.6963625	1.507592	13,554
	7	0.26578209	0.6668722	1.467029	12,980
	8	0.27260069	0.6839807	1.566918	13,313
	1	0.22298667	0.5594945	1.282096	10,890
5	2	0.13366914	0.7559931	1.377628	6528
	8	0.12396339	0.7011002	1.606137	6054
	7	0.12224338	0.6913723	1.520926	5970
	3	0.12199767	0.6899826	1.493780	5958

The used metrics are *Support, Confidence, Lift, Count*, and are defined as follows. Let *N* and *M* be two clusters. *Support({N}{M})* is the ratio of the number of transactions/tickets including *N* and *M* with respect to the total number of transactions. *Confidence({N}{M})* is the ratio of the number of transactions containing *N* and *M* with respect to the total number of transactions containing *N*. *Lift({N}{M})* is the ratio of confidence of *N* with respect to the total number of transactions containing *M. Count({N}{M})* is the number of transactions containing *N* or *M*. To generate the recommendations, we considered the top 5 clusters with highest *Support* and suggested one of the best-selling items (*Count*) within the cluster.

**item associated:** in order to increase the customer's purchase frequency, we generated suggestions on the basis of what has been purchased in the last three months. For the generation we have proceeded as follows: through association rules using a priori algorithm [1] we have defined pairs of items *(i,j)* with *Support* >  = *0.001* and *Confidence* >  = *0.01*. If a customer buys item *i* then item *j* will be suggested. This is the typical suggestion which can be delivered for stimulating the return on the shop. In order to take into account the evolution of the market and transactions the computation of Table 7 data, and thus of the association rules, is updated periodically. The periodic assessment has to take into account at least the last 6 months according to Table 5, which provides the evidence of transaction frequency/recency of customers.

**suggestions for serendipity:** randomly selecting items to be suggested from the whole present collection, taking also into account what is available in the physical shop.

### Considerations on Functional Dependencies

The above-described techniques for producing the suggestions are covering almost all cases. Recently, we have also analysed the usage of Functional Dependencies and their imprecise/relaxed and/or precise approaches [4, 12, 13, 27]. Those approaches are mainly focussed on identifying the complexity of relationships on data models. And thus, they can be profitably used to identify association rules, extracting the possible dependencies among the different fields related to user, products and transactions in a recommendation system. According to Section IV.D, for suggestions produced as *item associated*, specific products have to be suggested to the users; thus, the metrics of Table 7 identify the typical relationships among items bought by users and belonging to different clusters. Then, on the basis of the last item bought by a specific user it is possible to land on specific products by using the clusters identified by the association.

We explored the possibility of using Relaxed Functional Dependencies, RFD [13] in the context of recommendation. The first exploration has been on using RFD on a large data set with all features listed in the above clusters, using different time ranges (taking into account that in retails the returning period of user is large). Thus, incremental changes must be performed on long time range. An interesting result has been obtained by using RFD on the list of transactions annotated with the user information including: RFM_TRN_AvgAmount, RFM_TRN_DaysFrequency and RFM_TRN_DaysRecency, and by using product CONFIGURATION, MERCHANDISE_TYPOLOGY, and BRAND. The analysis has been limited to these features since all the other features have been discovered to be strongly dependent on them. The RFD has been produced by using the RFD-Discovery tool (https://github.com/dariodip/rfd-discovery) which is referring to [13], using a bottom-up approach. With this approach it has been possible to identify relevant dependencies, for example, on the basis of a given distance or similarity on:

- Average Spending, which may help to identify the products the user could be interested to. This is also modelled by the *customer similarity approach*, observing that users tend to spend the same budget in average
- MERCHANDISE_TYPOLOGY (using a semantic distance) which may help to identify typical user’s average spending, frequency, and recency. This last relationship could be used to identify a potentially similar cluster of users that could be prone to buy certain kind of products.

The distance adopted worked on numbers and on strings. For strings the tool exploited the lexical data base WordNet for computing the semantic distance.

Therefore, the RFD techniques could be used for computing association rules among the fields. Most of the associations are straight forward, and in most cases, they produce similar results than those of the above presented multi clustering approach, which also depend on multidimensional distances. The techniques for differentials or incremental estimation of FD can be also used to detect changes [11, 24]. In our approach the evolution of relationships on feature/clustering is progressively adapted by periodically recomputing the clusters and metrics of Table 7.

### Consuming Suggestions

The suggestions are provided to the Suggestion Table, which is structured as described in Table 8. With this table structure it is possible to save both generalized suggestions (e.g., by customer age or gender) and customer-identified suggestions.

**Table 8:** Suggestion Table Schema Description

Field name	*Description*
Recomm_id	Id suggestion
Customer_id	customer identification -1 for generalised suggestions
Type	contains information about the type of suggestion
Suggestion	item identifier to suggest (id or list)
Preferences	pattern of the item to be suggested (color, size, etc.)
Created	Suggestion creation date
Duration	Number of days the suggestion is valid
Date_Issued	Date in which it has been spent
How	Contains information on how the suggestion was submitted (via web or in-store)
Feedback	On Likert scale 1–5 (1 is good), or NULL
Interest	interest shown by the customer (tried on in the fitting room, purchased etc.)

Please note that the table does not provide all the information since the identified item ID allows to recover the description from the catalogue, and similarly the customer ID allows to recover the current status on the shop to avoid proposing multiple similar suggestions. Identical suggestions are also avoided since the Date_Issued marks when the suggestion has been spent. A segment of the Suggestion Table with instances is reported in Table 9.

**Table 9:** An example of the Suggestion Table status

Recomm_id	Customer_id	Type	Suggestion	Preferences	Created	Duration	Date_Issued	How	Feedback	Interest
…	…	C_SEREND	30,624	C = 4, T = 48	2020–09-18	60	2020–11-10	email	NULL	NULL
…	…	C_SEREND	10,799	…	2020–09-18	60	2020–10-02	WEB	1	Purchased
…	…	C_CORR	22,389	…	2020–09-18	60	2020–10-03	InStore	4	FittingRoom
…	.	C_COMPL	19,149	…	2020–09-18	60	2020–10-04	email	NULL	NULL
…	…	…	…	…	…	…	…	…	…	…

## Assessment and Validation

The recommendation system has been validated in a store located in Florence and on the online store as follows. We have exploited the data collected until December 2019 to test and tune the solution, verifying if the suggestions produced were also provided by the Assistant in shops and finally acquired by the customers. The algorithm updates the clusters monthly and generates the new suggestions daily. Considering the generated suggestions, without stimulating customers, we verified if there was a match among suggestions and items purchased by customers in the period January—June 2020, by checking transactions and verifying the shop assistants (which are the reference experts). This analysis showed that on about 400 customers who bought, about 10,000 suggestions were generated. On suggestions generated, the 6.36% items were purchased. This was considered the minimum level of reaching with the efficiency since resulted to be possible without the tool. Then, the recommendation system was tuned on operative modality from July 2020 until December 2020, to stimulate a certain class of users, entering in the store, using the totem in the store and by mail for ecommerce. This analysis with the stimulated customers showed that on 67 selected customers in the trial, 3050 suggestions have been generated, while only about the 20% has been actually sent to the customers (on shops and/or email). On the items suggested, the 9.84% of them were actually acquired or tested. Therefore, using the stimulus of the recommendation system, we have increased the customers’ attention of the 3.48%. The period for the assessment and validation was also complicated by the COVID-19 pandemic which strongly limited the access to the stores, and the validation via the e-commerce without the effective verification of the shop assistant is not comparable with the conditions of the 2019.

## Conclusions

In this paper, a recommendation system in the context of fashion retail has been proposed and described, relying on a multi-level clustering approach of items and users’ profiles in online and physical stores. The solution has been developed in the context of the Feedback project founded by Regione Toscana, and has been conducted on real retail company Tessilform, and it has been validated against real data from December 2019 to December 2020, showing that the use of the proposed recommendation tool generated stimulus to the customers which brought to an increase of buyers’ attention and purchase increase of 3.48%. The solutions proposed has demonstrated to be functional also in the presence of low number of customers and items (as happens in retails shops, in which the items are of high value), and when suggestions are mediated by the assistants, as happens in the fashion retail shops. Moreover, the proposed solution addresses and solved lacks and issues which are present in current state of the art tools, such as also the cold start problems in generating recommendations for newly acquired customers, since it relies on rules mining techniques, allowing to predict the purchase behaviour of new users. Our solution is also GDPR compliant, addressing the current strict policies for users’ data privacy, solving one of the main issues for managing users’ demographic details.
